# Systemic vasculitis in a child mimicking mercury poisoning

**DOI:** 10.1186/1546-0096-12-S1-P364

**Published:** 2014-09-17

**Authors:** Zübeyde Gündüz, Betül Sözeri, Ayşenur Paç Kısaarslan, Sibel Yel, Ruhan Düşünsel

**Affiliations:** 1 Pediatric Rheumatology, Erciyes University Faculty of Medicine, Kayseri, Turkey; 2 Pediatric Nephrology, Erciyes University Faculty of Medicine, Kayseri, Turkey

## Introduction

We reported a patient with mercury poisoning who presented as systemic vasculitis

## Results

A 12 year-old girl was admitted to our hospital with complaints of weakness, weight loss (10 kg in the last month), excessive sweating, and abdominal and joint pains. Her complaints started 6 weeks prior to admittance when she began to suffer from intermittent fever, acrodynia and back and abdominal pain. Also she was found to have red painful hands, a blood pressure 170/120 mm Hg, and tachycardia. In laboratory examinations, acute phase reactants were very high while viral markers, ANA and ANCA were negative. Renal doppler ultrasound and MR angiography were normal. We found sensorial neuropathy in her legs. Her blood pressure was controlled with 2 different antihypertensive medicines. The patient’s symptoms were not controlled with steroid treatment, we continued our research. A blood mercury concentration of 12.4Mcg/L (ref value 0,3 mcg/L )and spot urine mercury concentration of 18.8 Mcg/L (ref value 0.15 Mcg/L) were discovered. The girl was treated with metalcaptase 25mg/kg three times daily for 5 days, then every 12 hours for another 2 weeks (total duration, 19 days).

## Conclusion

Symptoms of mercury poisoning can vary greatly and mimic many acute diseases. Cardiovascular symptoms have been reported with acute poisoning. We conclude that inclusion of mercury intoxication in the differential diagnosis in vasculitis early on can help avoid unnecessary and invasive diagnostic tests and therapeutic interventions.

## Disclosure of interest

None declared

